# Solitary Fibrous Tumor of Nasal Cavity: A Case Report 

**Published:** 2015-07

**Authors:** George Ani Mathew, Gaurav Ashish, Amit Kumar Tyagi, Ramanathan Chandrashekharan, Roshna Rose Paul

**Affiliations:** 1 *Department of Otorhinolaryngology, Christian medical college ,Vellore ,Tamil Nadu, India.*

**Keywords:** CD 34, Immunohistochemistry, Lateral rhinotomy, Medial maxillectomy, Nasal cavity, Solitary fibrous tumour, Vimentin

## Abstract

**Introduction:**

Solitary fibrous tumours (SFTs) of the nose and paranasal sinuses are extremely rare. These were originally described as neoplasms of the pleura originating from spindle cells. It is further sub-classified as a benign type of mesothelial tumour. Its occurrence in many extra pleural sites have been reported earlier, mainly in the liver, parapharyngeal space, sublingual glands, tongue, parotid gland, thyroid, periorbital region, and very occasionally in the nose and paranasal sinus area.

**Case Report:**

A 28-year-old man with a 6 month history of persistent progressive left nasal obstruction and watering of the left eye is reported. Further imaging by CT and MRI revealed a large, left-sided, highly vascular, nasal cavity mass (Figs.1-4) pushing laterally on the medial wall of the maxilla. The patient underwent a lateral rhinotomy, which proceeded with the excision of the mass. Histopathological analysis of the specimen was consistent with SFT.

**Conclusion:**

This case is reported to develop insights regarding diagnosis and management of such rare tumours.

## Introduction

Solitary fibrous tumour (SFT), also known as benign fibrous mesothelioma or submesothelial fibroma (1), is sub-classified under existing mesothelial tumours. These tumours were initially thought to arise from the mesothelium and were named as local mesothelioma, however later studies showed them to arise from submesothelial fibroblast-like cells (2). It was first described in the pleura by Klemper and Rabin in 1931 and was later referred to as SFT of the pleura and peritoneum with absence of mesothelial differentiation (3,4). 

Various extra pleural sites have also been reported in literature; but its occurrence in the nose and paranasal sinuses is very rare. Adequate surgical excision with disease free tumour margin is usually curative. Around 27 such cases have been reported in world literature; however, the first case report of SFT found in the nose and paranasal sinuses seems to be from India.

## Case Report

A 28 year old man, with a 6 month history of progressive unilateral left-sided persistent nasal obstruction, watering of left eye, and intermittent epistaxis was presented. He had no other known co-morbidities or history of bleeding diathesis. 

**Fig 1:**
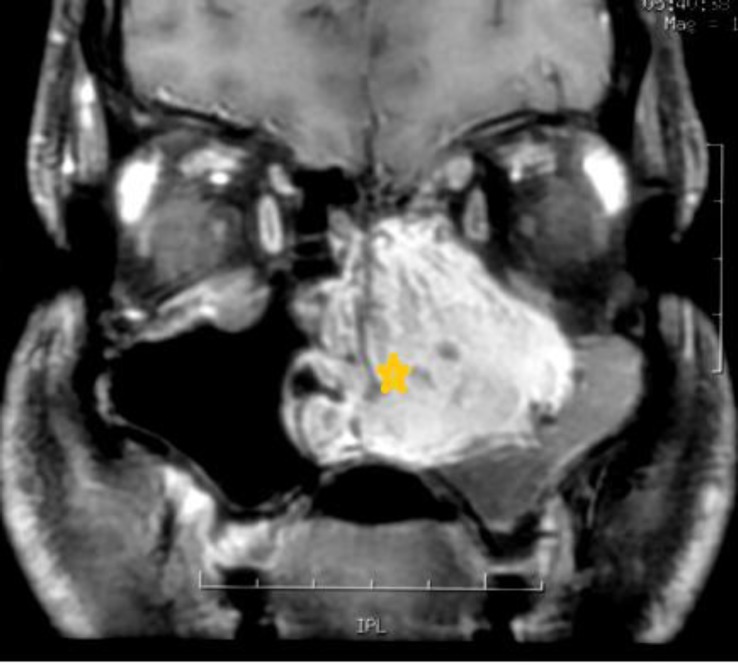
Tumour showing enhancement with contrast

**Fig 2:**
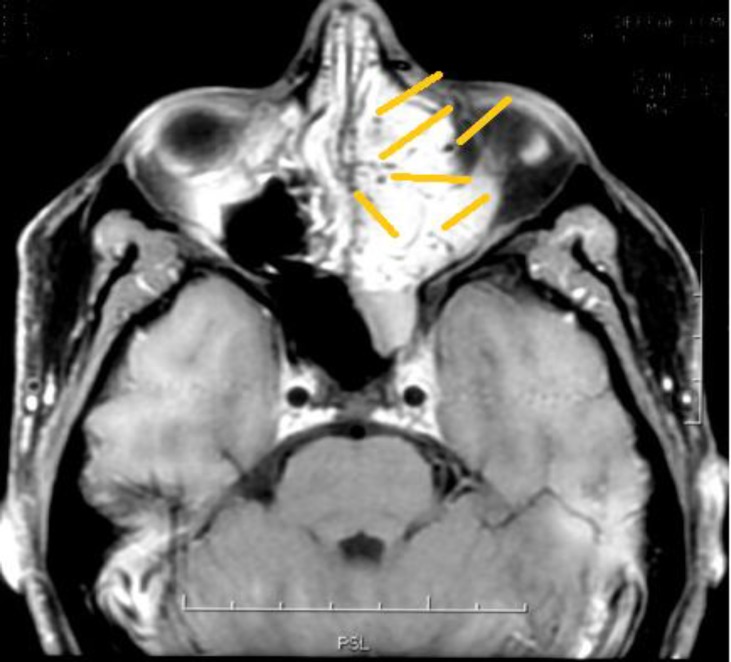
Tumour demonstrating flow voids indicating vascularity

**Fig 3:**
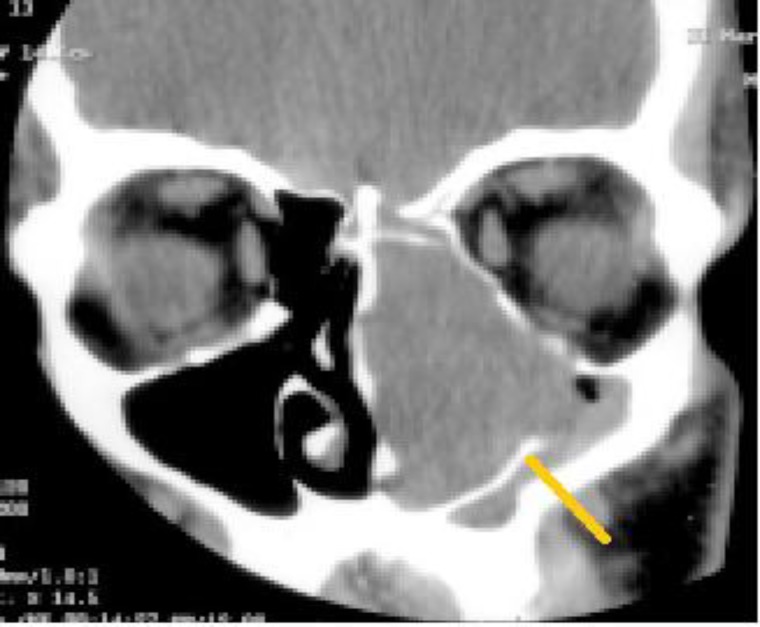
Tumour displacing the lateral wall laterally and inferiorly

**Fig 4:**
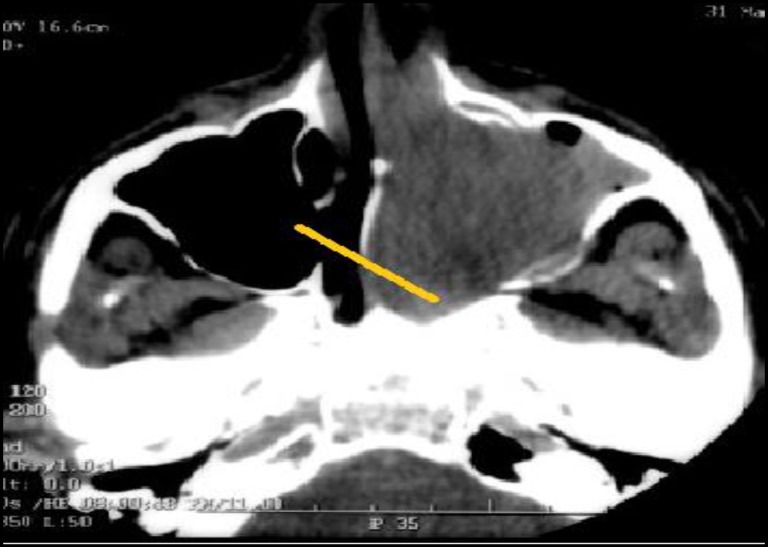
Tumour extending upto the nasopharynx

Rigid nasal endoscopy suggested a large mucosa covered mass filling the entire left nasal cavity. It was further evaluated with the help of Gadolinium-enhanced magnetic resonance imaging (MRI) of the brain and paranasal sinuses with computed tomography (CT) of the paranasal sinuses, which revealed a huge expansile enhancing mass, with multiple flow voids (Figs. 1,2) in the left nasal cavity extending upto the nasopharynx (Fig. 3), which was pushing on the left lateral nasal wall laterally (Fig. 4) and causing deviation of the nasal septum to the right.

**Fig 5:**
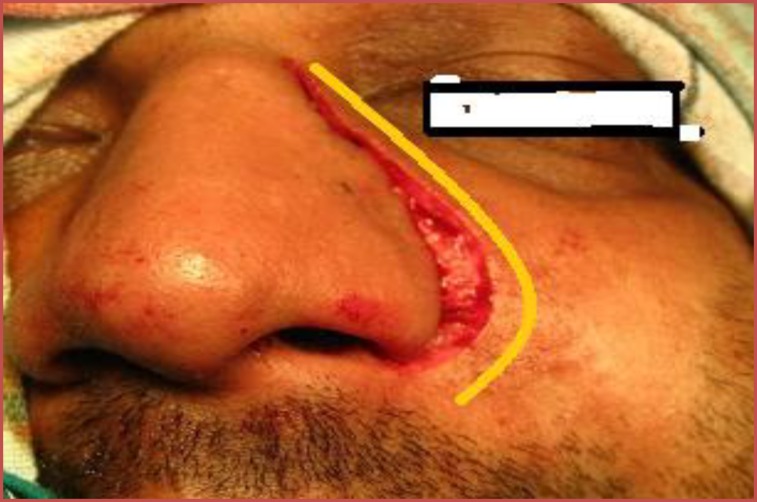
Lateral rhinotomy

Due to the highly vascular nature and extent of the tumour, the patient underwent a lateral rhinotomy (Fig.5), which proceeded with medial maxillectomy and piecemeal removal of the tumour. 

**Fig 6:**
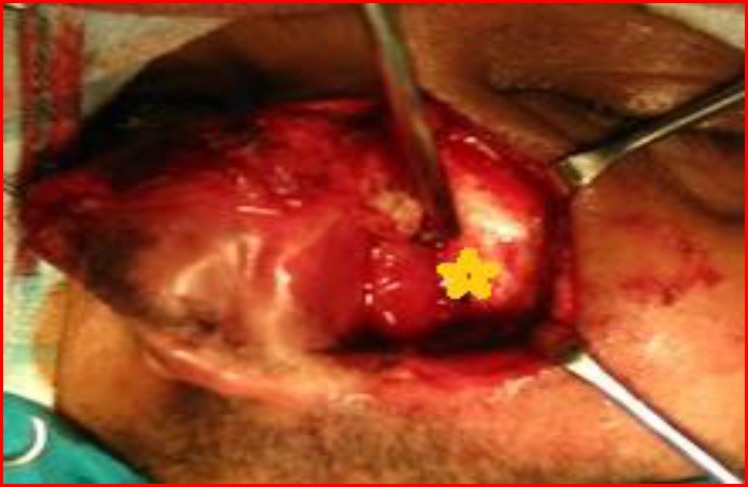
Depicting tumour in left nasal cavity

Intraoperative findings showed a soft to firm, vascular mass filling the left nasal cavity (Fig.6) and pushing the left lateral nasal wall laterally. It was also extending posteriorly upto the choana and pushing the nasal septum to the opposite side. Pre-operatively, severe bleeding was encountered as the tumour easily crumbled on manipulation. The tumour was removed entirely and haemostasis was achieved. The specimen was sent for histopathological examination. 

**Fig 7:**
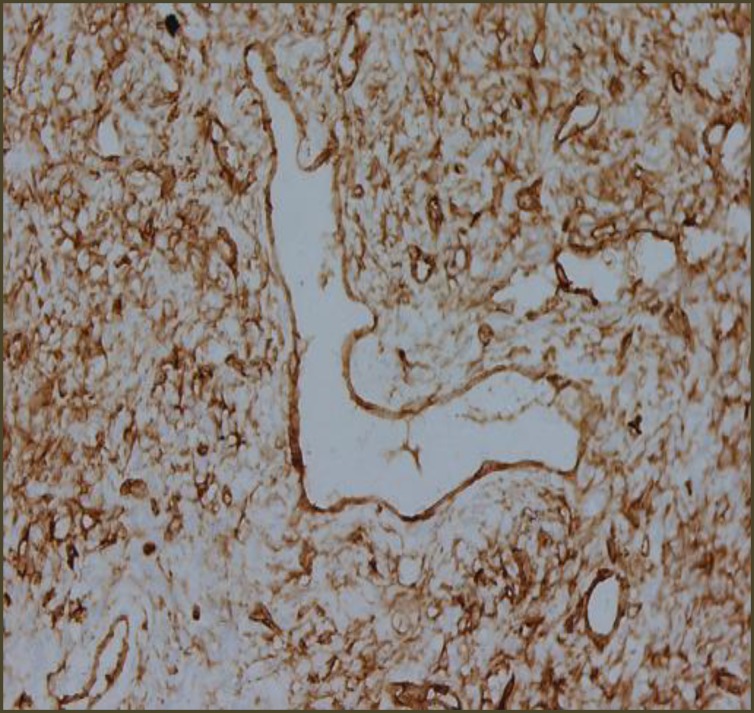
CD 34 positive

**Fig 8:**
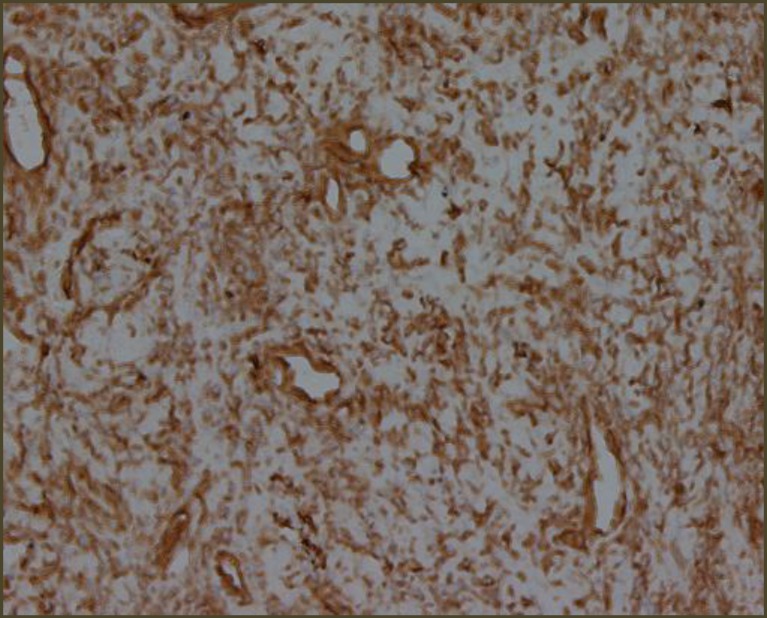
Vimentin positive

**Fig 9:**
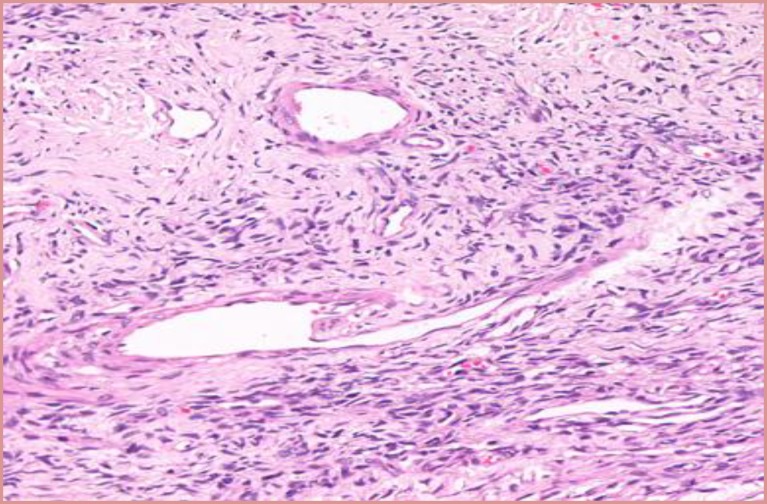
Fusiform cells with dense collagen stroma

**Fig 10:**
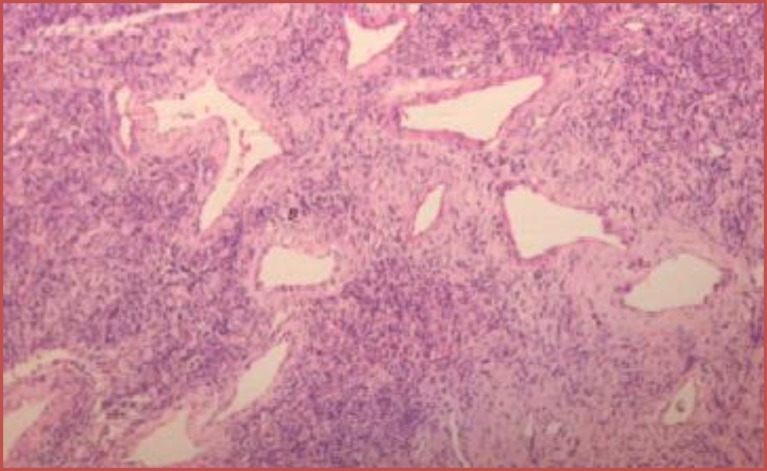
Ectatic vascular areas of varying diameter

**Fig 11:**
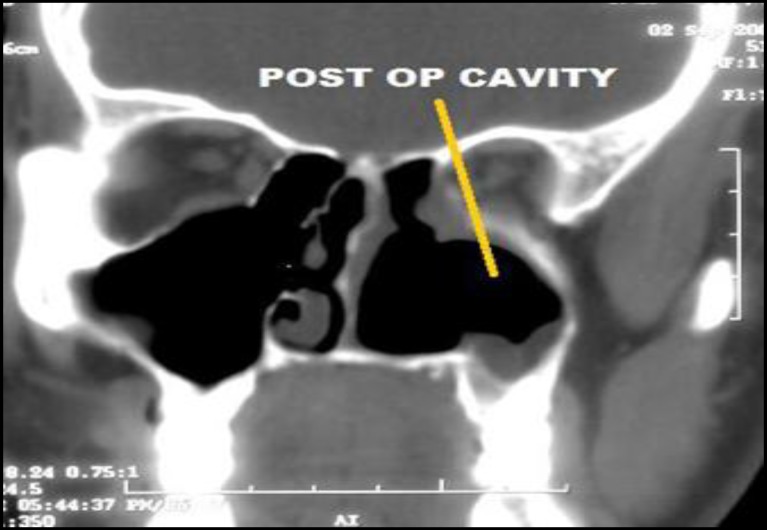
Post op imaging showing disease free status

**Fig 12:**
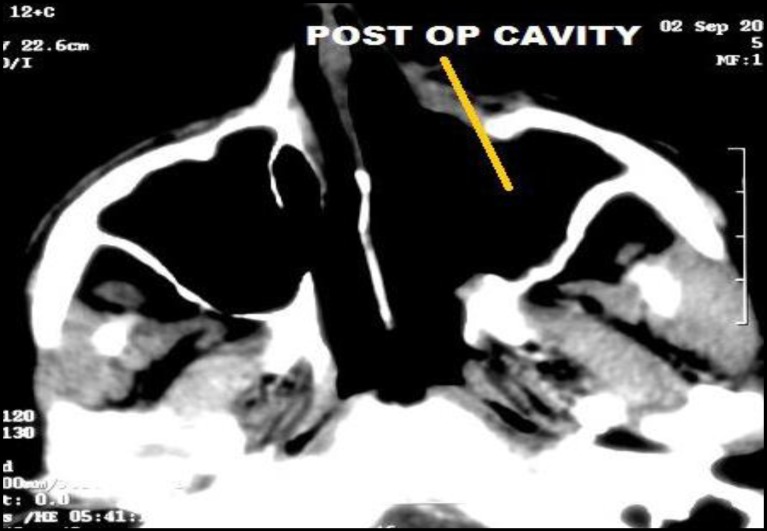
Post op imaging

Microscopic Examination revealed a hypo cellular neoplasm consisting of fusiform cells within a dense collagen stroma (Fig.7). There was several ectatic vascular areas of different sizes (Fig.8). No mitotic activity, necrosis, or nuclear pleomorphism was noticed in the tumour. Immunohisto- chemistry confirmed the diagnosis of SFT by demonstrating positivity for CD34 (Fig.9) and Vimentin (Fig. 10). The spindle cells were negative for AE1/AE3 Cytokeratin (epithelial marker), HHF35 (smooth muscle actin), and S100 protein (neural marker). Upon follow up, the patient progressed well postoperatively, with no recurrence three and a half years after surgery (Figs.11,12). 

## Discussion

SFT are rare tumors of mesothelial origin which generally occur in adults in the third to fourth decade of life; but the age of onset has been reported ranging from 9 to 86 years (5). 

Classically, these tumors were described to originate from the spindle cells of the pleura. However, various extra pleural sites have been reported, namely the liver, parapharyngeal space, sublingual gland, tongue, orbit, eyelids, nose, paranasal sinuses, orbit, parotid gland, thyroid, and laryngopharynx (6-8). 

Extrathoracic head and neck SFTs also tend to behave in a benign fashion, in a pattern similar to their intrathoracic counterparts (9). Nasal and extra-pleural SFTs are predominantly benign, in contrast with more aggressive behaviour found in 23% of pleural tumors. However, rarely have SFTs been associated with malignant transformation and none have been reported in the sinonasal region until now (10).

Sinonasal SFTs present themselves as a slow-growing painless mass.If symptomatic, the most common presentation of SFT is usually nasal obstruction, rhinorrhea, intermittent epistaxis, and exophthalmos. As per available literature, the size of the tumour usually ranges from 2.8 to 8cm in its major axis (11,12). 

These tumours are usually encapsulated, red, and fibrous. Here a soft to firm, well-encapsulated mass was observed. Non-contrast CT shows homogeneous isoattenuation compared with gray matter, and the tumours usually have marked enhancement after the administration of contrast material (12). 

Classically, on T2-weighted MRI, the soft-tissue component of SFTs is iso- to hypo-intense (13). These features were consistent with this case. 

Differential diagnosis of sinonasal SFT should include inverted papilloma, hemangioma, schwannomas, juvenile angiofibroma, angiomatous polyps, and hemangiopericytoma (14). 

Morphologically, SFT has a number of specific macroscopic, histologic and immunophenotypic characteristics that are pathognomonic and help the pathologist to make the correct diagnosis (15). Macroscopically, it can be seen as a pedicled or sessile encapsulated mass and histologically, it is composed of spindle cells arranged in a nonspecific pattern with varying vascularity, as was noted in this case. Presence of areas of hyalinization usually adjacent to collagen deposits is also a characteristic feature of SFT (9). 

The presence of CD34 and Vimentin positivity on immunohistochemical analysis is a typical finding and over half of all tumours are also CD99 positive (9). 

In addition, 75% of the tumours examined by Hasegawa and colleagues were positive for bcl-2 protein (9). 

Ultrastructural and immunohistochemical studies have suggested that SFTs primarily originate from mesenchymal fibroblast-like cells (16). The definitive diagnosis of SFT relies on the characteristic histopathological features and specific immunohistochemical markers (12). 

For SFTs of the nasal cavity endoscopic excision is the preferred surgical approach, although lateral rhinotomy, medial maxillectomy, external ethmoidectomy, and transfacial endoscopic approaches have been described (17-19). In this case, an open approach was preferred when considering the highly vascular nature of the tumour.

5 to 10% of all extra pleural SFTs have shown recurrence (14,20). Resectability is the most important prognostic factor. Therefore, effective treatment of SFTs of the nasal cavity and paranasal sinuses involves en bloc surgical excision (21). 

Prognosis mainly depends on the completeness of the surgical resection. However, the small number of SFT cases of the upper respiratory tract limits an estimation of accurate prognosis of extra pleural tumours and their clinical behaviour. 

## Conclusion

In conclusion, a rare case of extensive SFT of the nasal cavity removed using lateral rhinotomy approach was presented. This case highlights the importance of diagnosing SFTs of the nasal cavity and paranasal sinuses, as their management differs from other tumours. This case also stresses the importance of immuno-histochemical and histopathological features in the diagnosis of SFT.
